# Towards Biohybrid Lung Development—Fibronectin-Coating Bestows Hemocompatibility of Gas Exchange Hollow Fiber Membranes by Improving Flow-Resistant Endothelialization

**DOI:** 10.3390/membranes12010035

**Published:** 2021-12-27

**Authors:** Michael Pflaum, Sophie Jurmann, Katherina Katsirntaki, Marisa Mälzer, Axel Haverich, Bettina Wiegmann

**Affiliations:** 1 Department of Cardiothoracic, Transplantation, and Vascular Surgery, Hannover Medical School, Carl-Neuberg Str. 1, 30625 Hannover, Germany; Pflaum.Michael@mh-hannover.de (M.P.); sc.jurmann@t-online.de (S.J.); katsirntaki.katherina@mh-hannover.de (K.K.); maelzer.marisa@mh-hannover.de (M.M.); Haverich.Axel@mh-hannover.de (A.H.); 2 Lower Saxony Center for Biomedical Engineering Implant Research and Development (NIFE), Stadtfelddamm 34, 30625 Hannover, Germany; 3 BREATH (Biomedical Research in Endstage and Obstructive Lung Disease Hannover), German Center for Lung Research (DZL), Carl-Neuberg Str. 1, 30625 Hannover, Germany

**Keywords:** biohybrid lung, endothelialization, hemocompatible surface

## Abstract

To provide an alternative treatment option for patients with end-stage lung disease, we aim for biohybrid lung development (BHL) based on hollow fiber membrane (HFM) technology used in extracorporeal membrane oxygenators. For long-term BHL application, complete hemocompatibility of all blood-contacting surfaces is indispensable and can be achieved by their endothelialization. Indeed, albumin/heparin (AH) coated HFM enables initial endothelialization, but as inexplicable cell loss under flow conditions was seen, we assessed an alternative HFM coating using fibronectin (FN). Therefore, endothelial cell (EC) adherence and viability on both coated HFM were analyzed by fluorescence-based staining. Functional leukocyte and thrombocyte adhesion assays were performed to evaluate hemocompatibility, also in comparison to blood plasma coated HFM as a clinically relevant control. To assess monolayer resistance and EC behavior under clinically relevant flow conditions, a mock circulation setup was established, which also facilitates imitation of lung-disease specific blood gas settings. Besides quantification of flow-associated cell loss, endothelial responses towards external stimuli, like flow exposure or TNFα stimulation, were analyzed by qRT-PCR, focusing on inflammation, thrombus formation and extracellular matrix production. Under static conditions, both coated HFM enabled the generation of a viable, confluent, non-inflammatory and anti-thrombogenic monolayer. However, by means of homogenous FN coating, cell retention and physiologic gene regulation towards an improved hemocompatible-and extracellular matrix producing phenotype, was significantly superior compared to the inhomogeneous AH coating. In summary, our adaptable in-house FN coating secures the endothelial requirements for long-term BHL application and may promote monolayer establishment on all other blood contacting surfaces of the BHL (e.g., cannulae).

## 1. Introduction

For patients, suffering from end-stage lung diseases (ELD), lung transplantation (LTx) is still the only curative therapy option. However, due to the limited number of organ donors and strict requirements to consider lungs as transplantable, only a few and rigorously selected patients can be transplanted [1]. As bridge-to-transplantation extracorporeal membrane oxygenation (ECMO) has become an essential solution for temporary respiratory support, inter alia, in the event of an acute exacerbation of these lung diseases [2]. Here, patient’s blood is partly bypassed through an oxygenator, consisting of a multitude of gas exchange hollow fiber membranes (HFM), carrying the oxygen-rich gas mixture. Through diffusion across the fiber walls of the HFM, sufficient blood gas exchange is achieved. However, ECMO application is limited to a few weeks only, as insufficient hemocompatibility of all artificial blood-contacting surfaces results in the adhesion of proteins and subsequent thrombocytes, initiating the formation of blood clots, which significantly impede the blood flow through the device [3], thereby decreasing its gas exchange efficiency [4,5]. In order to minimize material-related thrombus formation, active and/or passive anti-coagulative coatings, e.g., albumin [6] and heparin [7], have been introduced onto these artificial surfaces. Furthermore, patients have to receive a meticulously controlled anti-coagulation therapy, which additionally bears a high risk for severe and lethal hemorrhage [4]. However, these measures can only delay but not prevent the above-mentioned adverse blood-material interactions until device failure becomes inevitable. It is clear that there is an urgent need to develop a long-lasting alternative to ECMO, which can be used as bridge-to-lung transplantation (LTx) and also as a final destination therapy, comparable to left ventricular assist devices for terminal heart failure [8].

Within recent years, several groups have followed different approaches to provide alternative therapy options for LTx, such as genetic editing of porcine lungs for xenogeneic transplantation, which need to escape hyperacute rejection [9], or the recellularization of decellularized lungs [10] from large animals or rather humans [11]. Furthermore, novel oxygenator devices based on microfluidics technology [12], as well as 3D printed lung-like structures are being developed [13]. However, these projects are still under development and have not yet been translated into clinical application.

Apart from these efforts, we aim for the development of the biohybrid lung (BHL), which has the potential to become a long-lasting pulmonary assist system as true alternative therapy option for LTx. The BHL is based on contemporary gas exchange HFM technology used in ECMO, but additionally holds the promise of obviating the material-induced thrombus formation and the concomitant device clogging, by establishing an endothelial cell monolayer on all blood-contacting surfaces. The main feature of this monolayer is to effectively shield off the foreign surface from proteins and thrombocytes, which otherwise would initiate the coagulation cascade. Further, endothelial proteins that reside on the endothelial cells (EC) will actively control hemostasis for optimized hemocompatibility [14]. For this approach, MHC silenced human cord blood derived ECs [15] and ECs derived from induced pluripotent stem cells (iPSC-ECs) [16] were identified as clinically relevant cell sources, which can be gained in sufficient numbers, while being immunologically invisible. Additional key findings within BHL development were the identification of potential surface coatings, enabling EC adhesion to the otherwise hydrophobic artificial material [17]. At this, promising results have been obtained with the commercially available and clinically used albumin/heparin (AH) coated HFM made of poly-4-methyl-1-pentene (PMP) [18], facilitating the establishment of a viable, confluent, non-activated, and non-thrombogenic EC monolayer [19]. Moreover, the monolayer remained stable under static cultivation conditions for up to 3 weeks [19] but indicating some cell loss under flow conditions [20]. However, it was noted that commercially applied AH coatings were not completely and uniformly dispersed all over the HFM surface, which may account for the occasionally observed detachment of cells under flow conditions, beside the fact that AH in not the physiologic substrate for ECs and fails to provide the potentially important homing cues. Moreover, gaps in the surface coating potentially may be a starting point for thrombus formation in the clinical setting. Alternatively, coatings using cyclic RGD-peptides [21] or titanium dioxide (TiO_2_) [17] were identified as also making gapless monolayer formation on HFM possible, but these respective coating techniques involved multiple cumbersome processing steps and potentially caused deterioration of the membrane, especially when transferring the technique from 2D film membranes to the clinically relevant 3D HFM. Furthermore, TiO_2_ coating in particular has been showed to adversely affect oxygen transfer [17].

Due to expedient features, fibronectin (FN) can also be used as HFM coating to mediate sufficient EC adhesion. As a well-known protein of the extracellular matrix, FN provides a physiologic basement membrane-like environment, which can be recognized by the cells through binding via integrins on the cell surface. Besides enabling the cultivation of various adhesion-dependent mammalian cells in basic research [22], integrin mediated signaling cascades could be necessary to ensure cell survival [23], especially during long-term application of the BHL. Furthermore, FN coating can be performed easily and reproducibly, enabling not only the application to the HFM, but also to all other blood-contacting surfaces of the BHL, such as housing and tubing. Recently, FN coating has also been suggested by other groups, inter alia, to mediate EC adhesion on 2D film membranes made of polydimethylsiloxane (PDMS) [24].

Accordingly, the aim of our study was to examine the suitability of our established independent, in-house protocol for effective FN coating of the 3D HFM in comparison to the commercially available AH coated 3D HFM to possibly identify an optimal alternative coating for BHL application. For this, we analyzed whether FN coating meets the requirement of promoting full hemocompatibility by the establishment of a confluent, viable, anti-thrombogenic, and non-inflammatory EC-monolayer on the HFM. This was tested by functional leukocyte and thrombocyte adhesion assays and compared to the clinically relevant positive control, mimicking the clinical scenario of HFM exposed to blood plasma. Additionally, we tested the capability of the ECs to express extracellular matrix proteins, as the formation of a basement membrane or glycocalyx-like matrix is beneficial for EC monolayer stability under flow conditions [25]. Finally, flow experiments were conducted, which were designed to assess monolayer stability under flow rates applicable in the animal model, with a view to approach BHL translation towards pre-clinical testing.

## 2. Materials and Methods

### 2.1. Endothelial Cell Isolation and Characterization

Endothelial Cells (ECs) were isolated from human umbilical cord blood as described previously [5]. Upon approval by, and in accordance with the local ethics board (approval number 9271_BO_K_2020), anonymized blood samples were collected directly after birth with informed consent of the mother. Mononuclear cells were seeded after density gradient centrifugation using Biocoll (Biochrom AG, Berlin, Germany) on standard tissue culture plastic (TCP) (Δsurface, nunc, Waltham, MA, USA) in EC growth medium EGM-2 (Lonza, Cologne, Germany), containing 10% fetal bovine serum (FBS) and incubated at 37 °C, 95% humidity and 5% CO_2_. Upon appearance of characteristic EC colonies, culture medium was changed to regular EGM-2 (Lonza, Cologne, Germany) containing 2% FBS. Following, ECs were detached with Trypsin/EDTA (0.05/0.02%, Biochrom AG, Berlin, Germany) when reaching 80% confluence and reseeded at a density of 8 × 10^3^ ECs/cm^2^ on TCP. Endothelial phenotype was confirmed by flow cytometry and RT-PCR for the presence of EC-specific markers, as described previously [18].

### 2.2. Hollow Fiber Membrane Preparation, Coating Assessment and Endothelialization

Within the following experiments, three different HFM, made of PMP, were analyzed. Therefore, commercially available AH coated HFM were taken from the iLA oxygenator (Novalung, Heilbronn, Germany), which is used as standard in the clinical setting. Second, uncoated HFM were obtained from Membrana GmbH (Wuppertal, Germany) and coated with FN using our in-house protocol by immersion in a fibronectin/Dulbecco’s phosphate buffered saline (DPBS, Gibco, Dreieich, Germany) solution (25 µg/mL) over night at 4 °C. All HFM were cropped to rectangular samples with a size of 3.3 × 1.8 cm [20].

In order to assess the uniformity of each coating, aforesaid HFM samples, as well as non-coated HFM, which were used as negative control (NC), were subjected to staining with 100 µM unspecific fluorescent protein dye 5-(and-6)-carboxy-tetramethylrhodamine succinimidyl ester (TAMRA) (Invitrogen, Eugene, OR, USA) in DPBS, at 37 °C for 20 min and analyzed under the fluorescence microscope (A1, Zeiss, Jena, Germany) equipped with a camera (AxioCam ICm1, Zeiss, Germany).

Following, AH coated and FN coated HFM were endothelialized, using the slightly modified protocol as described previously [19]. Briefly, two HFM samples were placed in 15 mL tubes, which were filled with EGM-2 medium containing 5 × 10^5^ ECs/mL and cultivated under rotation (1 rpm) for 4 h, at 37 °C. Thereafter, samples were sandwiched between two custom-made silicone frames and incubated for 48 h under static culture conditions. Then, qualitative and quantitative analyses of the endothelial monolayer on the HFM were performed as described below. Additionally, these endothelialized HFM were supplied on the one hand to biocompatibility analysis, in particular by means of functional leukocyte and thrombocyte adhesion assays and on the other hand to flow exposure experiments, which were assessed in the same qualitative and quantitative manner as the ECs under the above-mentioned static culture conditions.

### 2.3. Qualitative and Quantitative Analysis of the Endothelial Monolayer on HFM

#### 2.3.1. Cell Counting of Adherent ECs on HFM

In order to determine the number of adherent ECs, respective HFM samples were transferred into a 15 mL tube containing 4 mL Trypsin/EDTA. After enzymatic cell detachment for 5 min at 37 °C, enzyme activity was blocked by adding 10 mL DMEM/10% FBS, followed by centrifugation at 300× *g* for 5 min. Then, supernatant and HFM were removed to resuspend the sedimented ECs in 1 mL PBS to measure the cell concentration using the automated cell counter (CASY, Omni Lab Sciences, Bremen, Germany). These measured concentrations were set in relation to the respective HFM surface areas, which were calculated as described previously [19]: n × (2 rπh), where r is the radius (180 µm), h the length and n the number of each separate fiber within the 1.8 cm wide HFM.

#### 2.3.2. Cell Tracking Dye and Cell Viability Staining

Endothelial monolayer confluence on the HFM was assessed by 25 µM cell tracker green staining (Thermo Fisher Scientific, Dreieich, Germany) in serum-free EBM-2 for 45 min. In order to assess EC viability on AH and FN coated HFM, samples were stained for 30 min in EGM-2 containing the DNA-intercalating dye Höchst33342 (10 µg/mL) and 1 µg/mL calcein-am, or rather calcein red (Sigma-Aldrich, Taufkirchen, Germany), if ECs were already labeled with cell tracker green. Images were acquired using the Fluorescence-Stereomicroscope (StereoDiscovery V8, Zeiss, Jena, Germany) equipped with the camera (AxioCam ICm1, Zeiss, Germany).

#### 2.3.3. Immunofluorescence Detection of Endothelial-Specific Cell Junction Protein VE-Cadherin

To detect the intercellular adherence junction protein VE-cadherin, ECs on HFM were gently washed with Dulbecco’s Phosphate-Buffered Saline (DPBS) and fixed in 4% (*v*/*v*) paraformaldehyde for 15 min, at room temperature (RT). Following rinsing with Phosphate-Buffered Saline (PBS; *w*/*o* Ca^2+^/Mg^2+^), ECs were permeabilized and blocked for unspecific antibody binding by incubation with 0.25% Triton-X100, diluted in Tris-buffered saline supplemented with 5% (serum of the respective host of the secondary antibody) for 20 min at RT. Then, the samples were rinsed three times with PBS and incubated with primary anti-VE-cadherin antibody (diluted at 1:50 in 1% bovine serum albumin (BSA) in PBS *w*/*o* Ca^2+^/Mg^2+^; AbD Serotec, Puchheim, Germany), for 1 h at RT. After three washing steps, fluorescence-labeled secondary antibodies, goat anti-mouse Cy2 (Jackson ImmunoResearch, Cambridge, UK) were applied for 1 h at RT in the dark. Nuclei were stained with Höchst3334 dye (10 µg/mL) added to the secondary antibody incubation for the last 20 min. Samples incubated with isotype-matching antibodies were prepared as controls in parallel. After embedding in mounting medium (Immumount, Dako, Germany), microscopic images were obtained using the above-mentioned fluorescence microscope.

#### 2.3.4. Visualization of Extracellular Matrix Protein Collagen Type-IV

For EC-specific extracellular matrix protein collagen type-IV verification, samples were fixed with methanol at −20 °C for 20 min. After permeabilization and blocking as described above, primary anti-collagen-IV (clone CIV 22, Dako, Hamburg, Germany, diluted 1:30 in 1% BSA in PBS) antibodies were applied for 1 h at RT, the same for the subsequent secondary antibodies (donkey anti-rabbit Cy3), before the above-mentioned washing steps, nuclei staining and mounting were performed to take microscopic images using the above-mentioned fluorescence microscope.

To detect the ECs and their produced collagen-type IV, surrounding the three-dimensional HFM, samples were methanol-fixed and embedded in TissueTek^®^-O.C.T. compound (VWR, Hannover, Germany) on dry ice. Cross-sections were generated using a Cryo-microtome (Mikrom HM 560, Thermo Fisher Scientific, Dreieich, Germany) with a thickness of 16 µM and gently transferred on glass slides laminated with double-sided, transparent glue tape (300LSE, 70 µm thick, 3 M, Neuss, Germany) to prevent sample detachment during staining procedure. For visualization of collagen-type IV via Confocal Laser Scanning microscopy (CLSM SP8-system, Leica Microsystems, Wetzlar, Germany), 15 stack images in 1 µm steps were acquired along the z-axis for an ROI and were combined in a volume projection image using ImageJ (Z-project, maximum brightness).

### 2.4. Gene Expression Analysis by Realtime qRT-PCR

Gene expression analysis was performed following the protocol as described previously [18]. Briefly, RNA was isolated from ECs, either seeded on the HFM or on tissue culture plastic (TCP), using the NucleoSpin II Kit (Machery-Nagel, Düren, Germany) according to the manufacturer instructions. RNA was transcribed into cDNA using Random Hexamer Primers and the RevertAid H Minus First Strand cDNA Synthesis Kit (Fermentas, Germany). cDNA samples were diluted 1:10 with dH_2_O and assessed for the expression level of relevant genes via realtime qRT PCR applying the SYBR Green Mix^®^ (Abgene, Thermo Fisher, Waltham, MA, USA) and the specific primer pairs (see Supplementary Table S1). PCR reaction and signal detection were carried out using the PeqStar 96Q cycler (PeqLabs, Erlangen, Germany), the data was analyzed applying the ΔCt-Method using β-actin as housekeeping gene. Relative expression levels of the statically cultivated ECs on HFM were compared to ECs on TCP under the same standard culture conditions (95% humidity, 37 °C, 5% CO_2_), whereas relative expression levels of ECs after flow exposure were compared to those of ECs on HFM, cultivated statically under the same arterial cultivation conditions (37 °C, pO_2_: 65 mmHg, pH: 7.4) For deliberate EC activation, TNFα (10 ng/mL) was added to statically or dynamically cultivated samples, 6 h before RNA Isolation.

### 2.5. Functional Leukocyte Adhesion Assay

For the leukocyte adhesion assay HL-60 human promyelocytic leukaemia cells (ATCC, Manassas, VA, USA) were suspension-cultured in RPMI 1640 (VLE-RPMI 1640, Biochrom AG, Berlin, Germany) supplemented with 10% (*v*/*v*) FBS. Cell tracker red (25 µM) staining was applied, following the manufacturer’s instructions, to label HL-60 cells.

#### 2.5.1. Qualitative Analysis of Adherent Leukocytes by Fluorescence Microscopy

FN and AH coated, endothelialized HFM were mounted in bespoke frames, of which one sample group per HFM coating was stimulated with TNFα (10 ng/mL) for 6 h. Following, calcein-am was used as endothelial viability staining. In parallel, AH or FN coated, but non-endothelialized HFM samples were immersed in normal blood plasma (Precision BioLogic Inc., Dartmouth, NS, Canada) at 37 °C for 6 h to serve as clinically relevant positive control (PC), mimicking the result of the inevitable contact of the HFM and the circulating blood, which initiates inflammatory responses and thrombus formation in the clinical setting.

Subsequent, samples were transferred into 50 mL tubes filled with 50 mL EGM-2 and labeled HL-60 cells at a concentration of 4 × 10^5^/mL and incubated under slow rotation (1 rpm) for 1 h at 37 °C. Then, HFM were gently washed three times in DPBS and transferred into TCP dishes containing EGM-2. Images were taken at 6 random ROIs across the respective samples, facing top and bottom of the dish, using the aforementioned fluorescence microscope.

#### 2.5.2. Qualitative Analysis by Scanning Electron Microscopy

Directly after microscopy, two patches (0.8 cm diameter) were excised from each HFM using a skin biopsy punch cutter and forwarded to SEM-fixation buffer (1.5% formaldehyde, 1.5% glutaraldehyde, 150 mM HEPES, pH 7.3) for 10 min at RT. Following, patches were dehydrated during sequential immersion in a series of gradually increasing ethanol concentrations (25%, 50%, 70%, 90% and 99.9% ethanol in dH_2_O) for 10 min each. After critical point drying, patches were sputtered with gold and assessed using the scanning electron microscope (SEM, Philipps SEM 505, Amsterdam, The Netherlands).

### 2.6. Functional Thrombocyte Adhesion Assay

Human thrombocyte concentrates were obtained from anonymized healthy donors who undergo plateletpheresis at the blood bank of the Hannover Medical School, with informed consent and according to the regulation of the local ethic committee. The thrombocyte concentrate was stored in blood collection tubes containing Ethylendiaminetetraacetic acid (EDTA) (Sarstedt, Germany), complying with the standards for blood storage.

#### 2.6.1. Qualitative Analysis by Fluorescence Microscopy

The thrombocyte concentrate was diluted 1:1 with HEP-Buffer and centrifuged at 800× *g* for 15 min at RT without brakes. Sedimented platelets were gently rinsed with wash-buffer before resuspension in Tyrode’s Buffer. For labeling, calcein red (1 µM) was added for 30 min at 37 °C to the thrombocytes/Tyrode’s suspension. After centrifugation, sedimented platelets were washed twice with Tyrode’s buffer before being resuspended in Tyrode’s buffer containing 3 mg/mL bovine serum albumin (BSA). FN and AH coated, endothelialized HFM, with or without deliberate TNFα activation, were mounted in polycarbonate frames and stained for viable cells using calcein-am (1 µM). Afterwards, these HFM samples but also PC were transferred into 50 mL tubes containing EGM-2 medium and the labeled thrombocytes at a concentration of 1 × 10^8^/mL. After 1 h under rotation at 1 rpm at 37 °C, samples were removed, gently drawn through a large volume of DPBS and placed in a culture dish filled with DPBS to assess them under the fluorescence microscope.

#### 2.6.2. Qualitative Analysis by Scanning Electron Microscopy

As described above, two patches from each HFM were prepared and forwarded to qualitative SEM-analysis.

#### 2.6.3. Quantitative Analysis by Sudan Black B Staining

For thrombocyte adhesion assay using Sudan Black B dye (SBB), a previously reported protocol was adapted [26,27]. Therefore, thrombocyte concentrate was diluted 1:10 with HEP-Buffer (140 mM NaCl, 2.7 mM KCl, 3.8 mM HEPES, 1 mM EGTA, pH 7.4, 15 ng/mL Iloprost), incubated for 10 min at RT and centrifuged at 800× *g* for 10 min. The sedimented platelets were gently resuspended in 4% paraformaldehyde solution and allowed to fix for 10 min. After 1:1 dilution with PBS, fixed platelets were centrifuged at 800× *g* for 10 min. The sedimented platelets were resuspended in PBS and SBB (in 70% Ethanol) was added at a dilution of 1:20. After staining in SBB solution for 60 min at RT, platelets were centrifuged and washed three times to delete residual SBB dye. FN and AH coated, endothelialized HFM, with or without deliberate TNFα activation for 6 h, also PCs were transferred in 15 mL tubes, containing cell medium and 1.33 × 10^7^/cm^2^ SBB-stained thrombocytes, which remained in the sample solution for 30 min while agitated on a rocking platform. Following incubation, all HFM samples were removed and drawn through a large volume of DPBS (500 mL, with Ca^2+^/Mg^2+^) to delete unbound platelets, and placed into new tubes containing 1 mL DMSO, to dissolve SBB from the adherent platelets. After 30 min SBB-containing DMSO was transferred into the wells of a 96 well plate in duplicates to measure the absorbance at 595 nm. Serial dilutions of a known numbers of SBB-stained platelets in DMSO were measured in parallel as reference.

### 2.7. Flow Exposure of Endothelialized HFM

Following static cultivation, AH, or rather FN coated, endothelialized HFM were mounted in the bespoke flow chamber as described previously [20]. For flow experiments, EGM-2 medium was taken from the stirred-tank reservoir (BioStat^®^, Sartorius, Göttingen, Germany) and applied by the peristaltic pump (MCP Ismatec, Cole-Parmer GmbH, Wertheim, Germany) to the three flow chambers running in parallel. The BioStat^®^ system was set to automatically adjust the oxygen concentration and pH of the EGM-2 to arterial blood gas levels, comparable to the future clinical setting of BHL application (pO_2_: 65 mmHg, pH: 7.4). Gas saturation levels were monitored on-line by the BioStat-system, single-point measurements were sampled at the outlet of each flow chamber hourly over the first 6 h and before completing the experiment after 24 h and analyzed for pO_2_, pCO_2_, pH, glucose and lactate using the blood gas analyzer (ABL90Flex, Radiometer, Krefeld, Germany). To gently adapt the endothelial monolayer on the respective HFM to flow conditions, the initial flow rate of 2 mL/min was doubled every 30 min up to 2 h, until the maximum of 15 mL/min, was reached, which was applied for the following 22 h. This flow rate equals the needed 30% cardiac output from real-scenario ECMO flow rates (1.5 L/min) [28] for BHL application, when downscaling to the custom-made flow chamber used in the small animal model [20]. A schematic view of the experimental setup for the flow application can be found in Supplementary Materials Figure S3.

### 2.8. Statistics

Three independent samples per group were analyzed, unless stated otherwise. All results were expressed as means with standard deviation (SD). For statistical analysis between two groups, unpaired, two-tailed *t*-test was applied, one-way ANOVA followed by Tukey’s Multiple Comparison Test (Prism Version 9, Graphpad Sotware Inc, San Diego, CA, USA) was used to find statistically significant differences between multiple groups. Statistical significance was indicated with * for *p* < 0.05, ** for *p* < 0.01, and *** for *p* < 0.001.

## 3. Results

### 3.1. Effective FN Coating of HFM Facilitates the Formation of a Viable and Confluent Endothelial Monolayer

**Figure 1:**
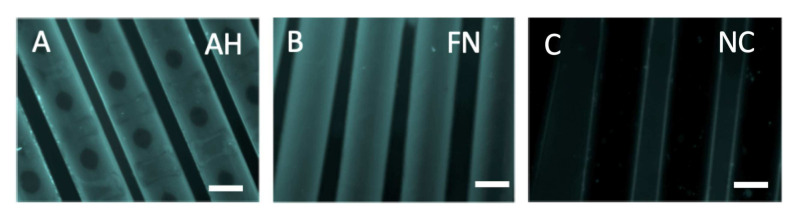
Analysis of coating distribution on HFM. TAMRA staining (**cyan**) was used to visualize the coating efficiency of AH coated (**A**), FN coated (**B**) and non-coated HFM (**C**) Scale: 250 µm.

**Figure 2:**
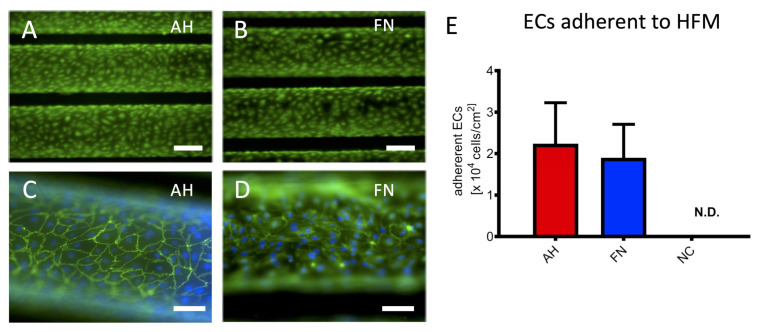
Viability, confluence and quantification of ECs adherent to HFM. Viable ECs on AH (**A**) and FN (**B**) coated HFM were visualized using calcein staining (**green**); Scale: 200 µm, VE-cadherin (**green**) and Hoechst (**blue**) indicated intercellular connection between ECs on AH (**C**) and FN (**D**) coated HFM; Scale: 100 µm. (**E**) Quantification of adherent ECs on AH and FN coated HFM, ECs on non-coated (NC) HFM were not detectable (N.D.).

Staining with unspecific fluorescent protein dye visualized regions on AH coated HFM with low or rather missing signal intensity (Figure 1A), whereas our established in-house protocol for FN coating resulted in the homogenous distribution over the entire HFM (Figure 1B). In contrast, no signal was detected on the surface of the non-coated HFM (NC). Fluorescence microscopy images revealed that both, AH (Figure 2A) and FN coated HFM (Figure 2B), enabled the generation of a viable and confluent endothelial monolayer, comprising ECs, which were interconnected via the cell junctional protein VE-cadherin (Figure 2C,D). With regard to the cellular quantification of adherent ECs in relation to the respective three-dimensional HFM surface area, cell counting revealed no statistically significant differences, as ECs on AH coated HFM (2.24 ± 0.99 × 10^4^ ECs/cm^2^) were comparable to adherent ECs on FN coated HFM (1.9 ± 0.81 × 10^4^ ECs/cm^2^, *p* > 0.05) (Figure 2E). The number of adherent ECs on non-coated HFM was below the detection level, therefore no fluorescence images were shown.

### 3.2. EC Monolayer on FN Coated HFM Shows Physiological and Hemocomatible Behavior

#### 3.2.1. qRT-PCR Confirms Anti-Thrombogenic and Non-Inflammatory Endothelial Status

**Figure 3:**
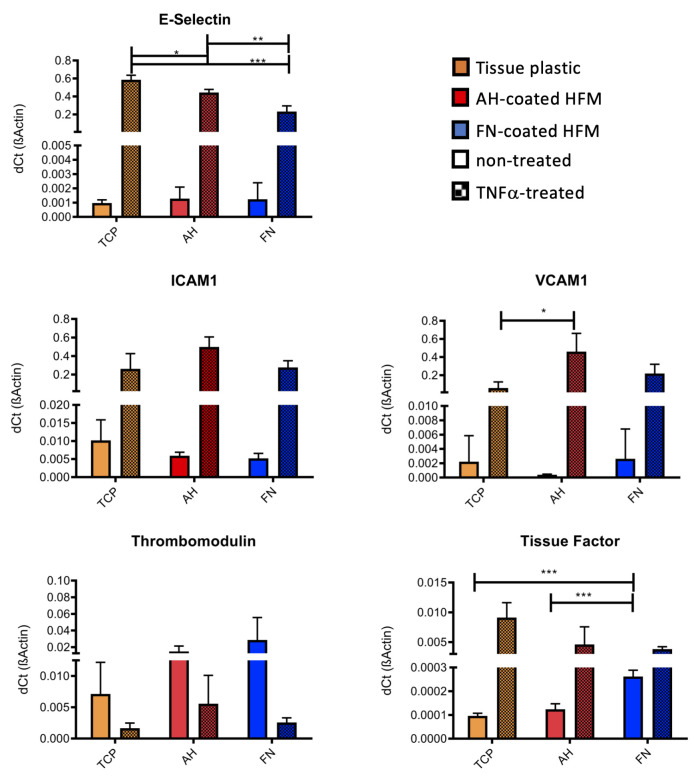
Gene expression analysis for EC activation state detection by realtime qRT PCR. Expression levels of endothelial specific activation markers of ECs cultivated on TCP, AH and FN coated HFM with and without TNFα treatment (* *p* < 0.05, ** *p* < 0.01, *** *p* < 0.001).

To assess, if AH, or rather FN coating had an impact on the physiologic anti-thrombogenic and non-inflammatory status of the seeded ECs, expression levels of EC-specific genes, which are associated with activation and thrombogenesis, were analyzed (Figure 3). No significant differences in the expression levels of the pro-inflammatory E-Selectin, VCAM1 and ICAM1 could be detected for unstimulated ECs cultivated on TCP compared to ECs on AH and FN coated HFM. Further, anti-thrombogenic thrombomodulin presented the highest expression levels in the non-stimulated ECs on FN coated HFM. In contrast, a significant but negligible increase in the expression level of tissue factor could also be seen for these unstimulated ECs compared to ECs on TCP and AH coated HFM. In general, endothelial activation status could be physiologically induced on all surfaces, as detected by the respective significant increases of the expression levels of E-Selectin, VCAM1, ICAM1 and tissue factor after TNFα stimulation and in comparison to the respective unstimulated ECs of each group. However, in detail the expression level of E-Selectin was significantly higher for TNFα-stimulated ECs on TCP and on AH coated HFM compared to FN coated HFM, presenting the lowest expression level.

As expected and physiologically appropriate, thrombomodulin gene expression was consistently lower in ECs on all substrates (TCP < AH < FN) upon deliberate cell activation compared to the unstimulated ECs. With respect to pro-thrombogenic tissue factor, gene expression analysis revealed both the lowest concentration and also the lowest increase after TNFα stimulation for ECs on FN coated HFM.

#### 3.2.2. Functional Leukocyte Adhesion Assay Approved Non-Inflammatory Endothelial Status

**Figure 4:**
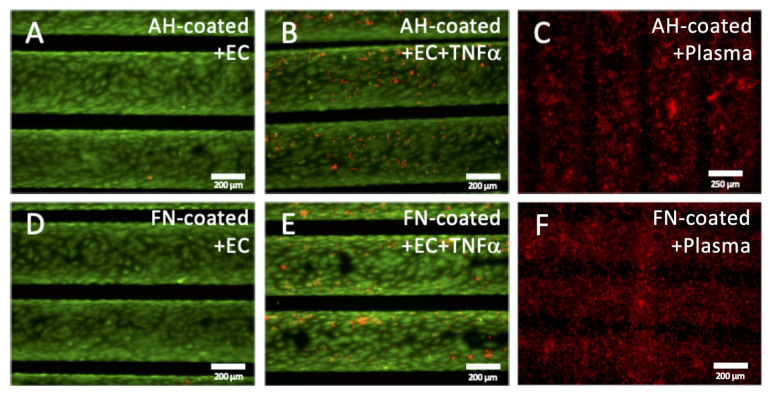
Leukocyte adhesion assay. Untreated (**A**,**D**), or rather TNFα stimulated (**B**,**E**) EC monolayers (**green**) on AH (**A**,**B**) and FN (**D**,**E**) coated HFM, also non-endothelialized AH (**C**) and FN coated HFM (**F**) after blood plasma immersion were incubated with HL-60 leukocytes (**red**); Scale: 200 µm.

Gene expression analyses were complemented by the functional leukocyte adhesion assay to prove the physiologic non-inflammatory status of the EC monolayer on HFM, additionally. Fluorescence microscopy detected only a few to zero adherent HL-60 cells on the monolayer of AH and FN coated HFM (Figure 4A,D). A marginal increase of adherent leukocytes could be seen on TNFα stimulated ECs on both coated HFM, though slightly more on AH coated HFM consistent with generally higher expression levels of the adhesion molecules E-Selectin, ICAM1 and VCAM1 (Figure 3 and Figure 4B,E). In contrast to the endothelialized HFM, independent of the respective coating, a prominent and apparent significantly higher number of adherent leukocytes were detected on both PCs (Figure 4C,F). Subsequent sample analysis using SEM imaging (Supplementary Materials Figure S1A–E) confirmed the results of the fluorescence-based assay, as the frequency of leukocytes was rarely detectable on AH coated and FN coated HFM (Supplementary Materials Figure S1A,D), compared to higher numbers of adherent leukocytes on the respective TNFα stimulated ECs (Supplementary Materials Figure S1B,E). Here again, the highest number of adherent leukocytes could be identified on both PCs (Supplementary Materials Figure S1C,F).

#### 3.2.3. Functional Thrombocyte Adhesion Assay Approves Anti-Thrombogenic Endothelial Status

**Figure 5:**
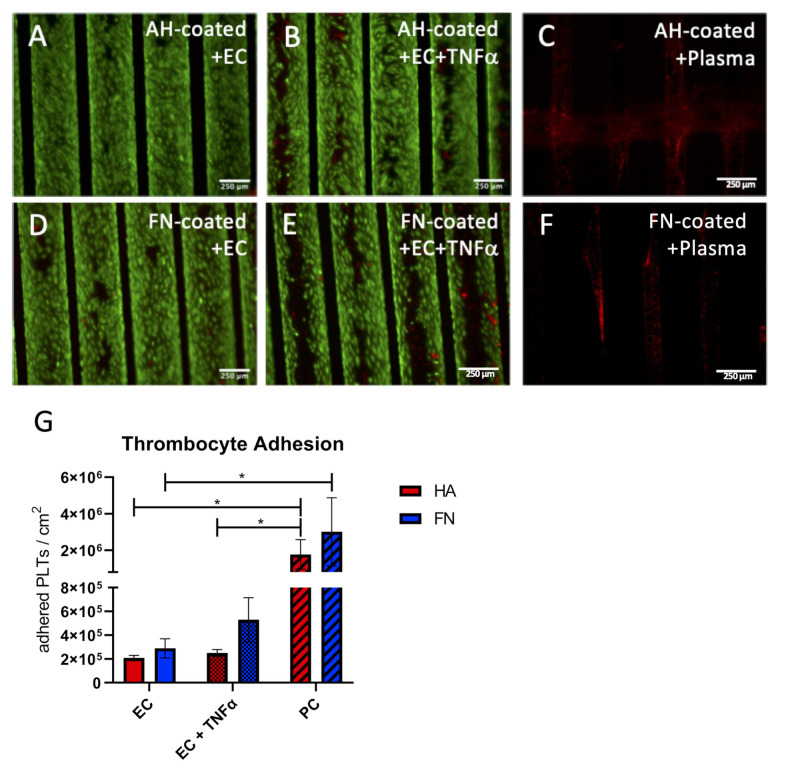
Platelet adhesion assay. For qualitative analysis, untreated (**A**,**D**), or rather TNFα stimulated (**B**,**E**) EC monolayers (green) on AH (**A**,**B**) and FN (**D**,**E**) coated HFM, also non-endothelialized AH (**C**) and FN coated HFM (**F**) after blood plasma immersion were incubated with thrombocytes (**red**); Scale: 250 µm. (**G**) Quantification of adherent thrombocytes on the respective endothelialized or non-seeded positive control (PC) HFM (*: *p* < 0.05).

Likewise, the functional thrombocyte adhesion assay approved the physiologic anti-thrombogenic status of the EC monolayer. Images obtained by fluorescence microscopy (Figure 4A–F) and SEM (Supplementary Materials Figure S2A–F) confirmed comparable results as seen in the leukocyte adhesion assay. In particular, only a few to zero adherent thrombocytes could be detected on the endothelial monolayer of AH and FN coated HFM (Figure 5A,D). By comparison, a marginal increase of adherent thrombocytes could be seen on TNFα stimulated ECs on both coated HFM. Moreover, independent of the respective coating, a prominent and apparent significantly higher number of adherent thrombocytes was identified on both PCs (Figure 5C,F). Quantitative analysis by SBB proved these findings, as the statistically highest number of adherent thrombocytes could be seen on both PC (FN coated PC 3.01 × 10^6^ ± 1.86 × 10^6^/cm^2^; AH coated HFM 1.77 × 10^6^ ± 8 × 10^5^/cm^2^) (Figure 5G). A slightly, but not statistically significant higher number of adherent thrombocytes presented for the TNFα stimulated monolayers compared to the unstimulated ones (AH HFM ECs vs. AH HFM ECs+TNFα: 2.08 × 10^5^ ± 2.20 × 10^4^/cm^2^ vs. 2.50 × 10^5^ 2.88 × 10^4^/cm^2^, and FN HFM ECs vs. FN HFM ECs+TNFα: 2.89 × 10^5^ ± 8.06 × 10^4^/cm^2^ vs. 5.30 × 10^5^ ± 1.85 × 10^5^).

### 3.3. Endothelial Monolayer on FN Coated HFM Physiologically Respond and Resist to Clinically Relevant Flow Conditions

#### 3.3.1. Endothelial Monolayer Resists the Applied Flow Conditions for 24 h under Stable Arterial Conditions

**Figure 6:**
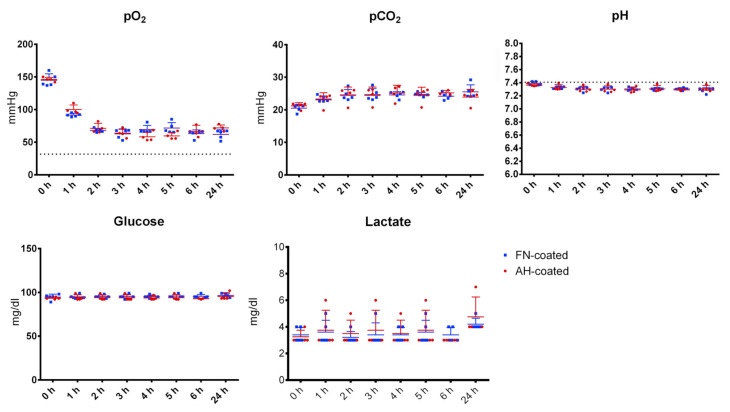
Medium gas analysis during flow exposure. Single point measurements of pO_2_, pCO_2_, pH, glucose, and lactate concentration during flow experiments with endothelialized AH (**red**) and FN (**blue**) coated HFM.

Within flow exposure experiments a stepwise increase of the flow rate in the first two hours was performed, at which the pO_2_ decreased steadily for both coated and endothelialized HFM without any significant difference (AH coated 146 ± 2.83 mmHg to 71.23 ± 6.62 mmHg, FN coated 147 ± 9.52 mmHg to 73.07 ± 11.69 mmHg) until reaching a stable plateau of around 68.12 mmHg until the end of the experiment. Levels of pCO_2_ developed opposed, from their starting values (AH coated 21.20 ± 0.87 mmHg, FN coated 20.28 ± 1.66 mmHg) up to the stable plateau around 24.7 mmHg after two h. During all experiments (*n* = 4), and irrespective of the coating type, pH, glucose and lactate values did not change significantly throughout the flow exposure time (Figure 6).

**Figure 7:**
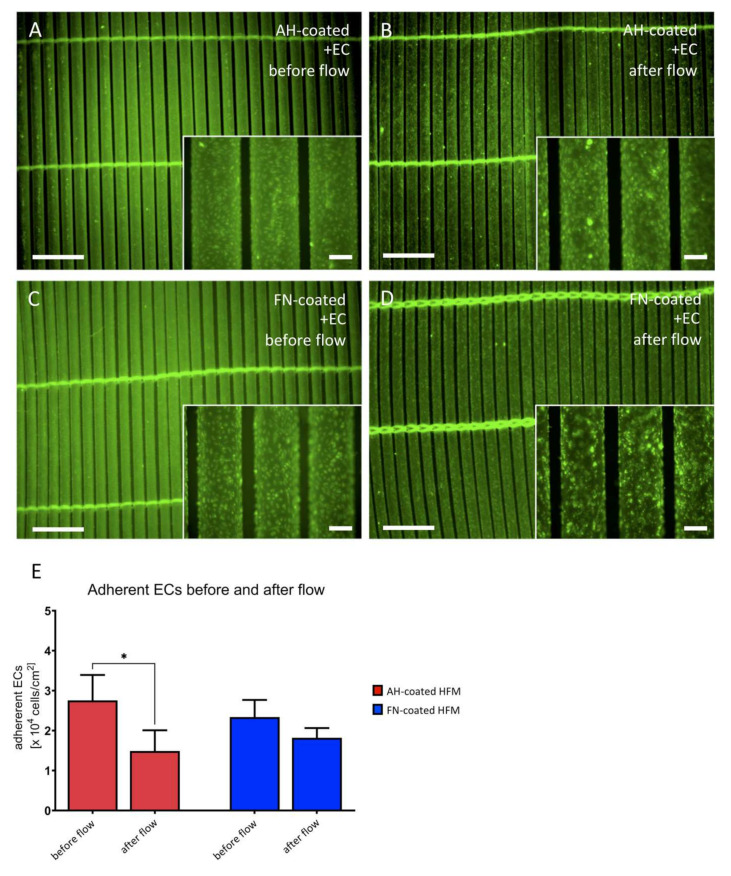
Assessment of EC monolayer after flow exposure. Labeled EC monolayers (**green**) on AH (**A**,**B**) or FN coated HFM (**C**,**D**) were assessed before (**A**,**C**) and after (**B**,**D**) flow exposure (lower magnification images (**A**–**D**) scale: 1.5 mm, inserted box (**A**–**D**): enlarged area of respective EC monolayer areas; Scale: 200 µm). (**E**) Quantification of adherent ECs on AH and FN coated HFM before and after flow exposure.

In order to assess endothelial flow stability on both coated HFM, fluorescence microscopic images were obtained directly before and after flow exposure (Figure 7A–D), indicating complete EC coverage of the HFM prior to flow application (Figure 7A,C), while few areas with decreased cell density could be observed on AH HFM after flow (Figure 7B). EC coverage on FN HFM after flow seemed to be less affected by flow exposure (Figure 7D).

In contrast, a significant cell loss was shown for ECs on AH coated HFM (prior to flow exposure: 1.49 ± 0.52 × 10^4^ ECs/cm^2^ vs. after flow exposure: 2.76 ± 0.64 × 10^4^ ECs/cm^2^, *p* < 0.05), while no statistically significant difference with the respect to the ECs on FN coated HFM could be detected (prior flow exposure 2.34 ± 0.43 × 10^4^ ECs/cm^2^ vs. after flow exposure: 1.82 ± 0.25 × 10^4^ ECs/cm^2^, *p* > 0.05) (Figure 7E).

#### 3.3.2. Endothelial Monolayer Preserves Non-Activated Status and Expresses Extracellular Matrix Proteins

**Figure 8:**
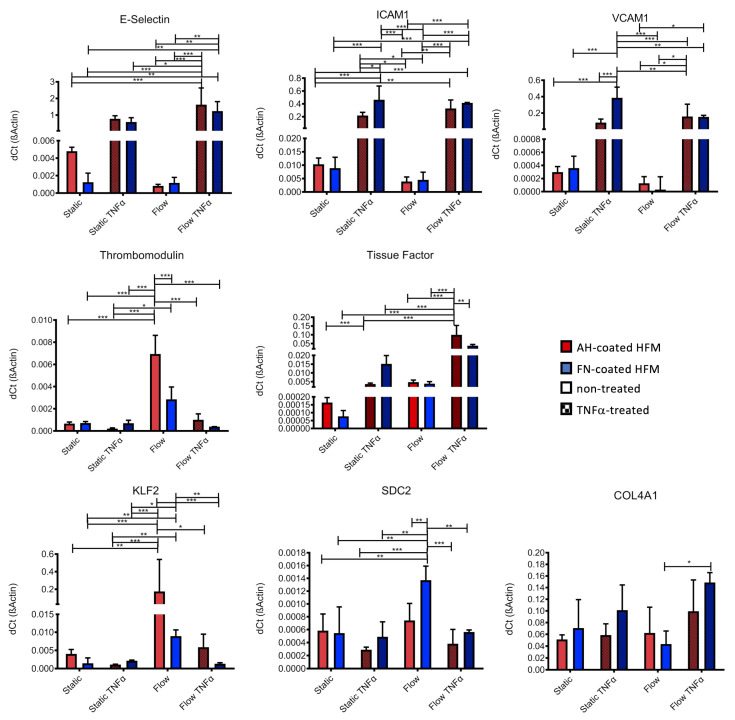
Gene expression analysis for EC activation state detection and the regulation of extracellular matrix components under flow conditions by realtime qRT-PCR. Expression levels were compared between ECs on both coated HFM under static conditions and under flow exposure, and with or without TNFα treatment (* *p* < 0.05, ** *p* < 0.01, *** *p* < 0.001).

EC monolayers established on both coated HFM with and without TNFα stimulation were analyzed and compared to statically cultivated ECs (*n* = 4) using qRT-PCR, relative expression levels of genes, which are indicative for cell activation in response to shear stress (Figure 8). As already seen in Figure 3, TNFα stimulation resulted in increased expression levels of all activation state markers (E-Selectin, ICAM1, VCAM1), though no major changes in gene expression levels of ECs under static cultivation to flow exposure could be detected. Thrombomodulin expression was significantly upregulated in ECs on AH coated HFM after flow exposure compared to the ECs on the FN coated HFM. Upon activation with TNFα under flow conditions, the expression level dropped again to the level detected under static conditions. Although a higher mean value for the expression of thrombomodulin was detected in flow-exposed ECs on FN coated HFM compared to the expression level under static conditions, the difference was not statistically significant. With regard to tissue factor, comparable levels were obtained for deliberately activated ECs under static condition, also for ECs on both coated HFM types after flow exposure. However, a significantly higher expression for tissue factor on both coatings was detected when flow-exposed ECs were additionally activated with TNFα.

KLF2, a known indicator for shear stress response, was significantly upregulated on both coating types only under flow conditions. After additional TNFα activation under flow conditions, KLF2 expression levels dropped back to levels that were detected under static conditions.

In order analyze the expression of extracellular matrix proteins, syndecan-2 (SDC2) gene expression level was analyzed as an indicative marker for glycocalyx production on the blood-facing side of the EC monolayer, also collagen-type IV Subunit A1 (COL4A1) as endothelium specific basement membrane matrix marker. Statistically significant highest expression levels for SDC2 could be seen for flow-exposed ECs on FN coated HFM compared to all other groups. COL4A1 gene activity was detected under all conditions and coatings, however a slightly and significantly higher expression was only found for flow exposed ECs on FN coated HFM with TNFα stimulation.

#### 3.3.3. Immunofluorescence Stainings Indicate Extracellular Matrix Protein Collagen Type-IV Synthesis by Endothelial Cells

**Figure 9:**
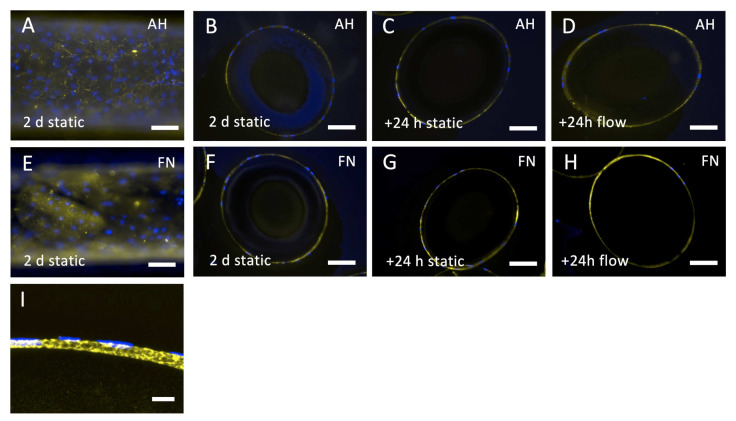
Collagen Type-IV detection on endothelialized and flow exposed HFM. Endothelialized AH (**A**–**D**,**I**) or FN (**E**–**H**) coated HFM were analyzed for collagen-type IV expression (yellow). Top view of endothelialized AH (**A**) and FN (**E**) coated HFM after 2 days under static cultivation. Ultrathin cross-sections of endothelialized AH (**B**) and FN (**F**) coated HFM after 2 days under static cultivation, after another 24 h either under static (**C**,**G**), or flow conditions (**D**,**H**). Höchst33342 dye was used to counterstain cell nuclei (**blue**); Scale: 100 µm. (**I**) Confocal laser scanning microscopy images were acquired to verify the basal-lamina-like location of collagen-type IV on the HFM surface; Scale: 10 µm.

Gene expression analysis revealed that COL4A1 was constantly expressed by ECs seeded on HFM either coated with AH or FN, independently of flow exposure. Using immunofluorescence staining and established cross-sectioning technique, it was possible to identify the correlation of the genetic activity to the expression of collagen type-IV as an important basement membrane protein, in particular around the whole circumference of each single fiber, especially for ECs on FN coated HFM. Collagen-type IV was detectable on the surfaces of all coated and endothelialized HFM (Figure 9A–H), as selected samples, assessed with confocal laser scanning microscopy, confirmed the location of the collagen-type IV signal between the HFM surface and the endothelial cell nuclei (Figure 9I). Of note was that the collagen-type IV matrix appeared rough and patchy on AH coated HFM, whereas FN coated HFM presented a smooth and homogenous deposition. While the signal was faint but present around the complete circumference of the HFM 2 days after of static cultivation (Figure 9A,B,E,F), signal intensity appeared more prominent around the fibers, which were cultivated for another 24 h under static (Figure 9C,G) or rather dynamic conditions (Figure 9D,H), indicating a more time-dependent, than flow-dependent synthesis.

## 4. Discussion

Our general strategy for BHL development is based on tissue engineering, which means that all artificial blood contacting surfaces of the ECMO will be endothelialized, to thereby shield the pro-thrombogenic artificial interfaces, as the endothelial monolayer presents specific surface proteins, e.g., thrombomodulin, which actively control blood stasis and inhibit thrombus formation [29]. However, due to the hydrophobic surface nature of these artificial materials, in particular of the HFM, their cell binding properties need to be improved, to achieve efficient EC adhesion, e.g., by proteins or peptides [21,30], TiO_2_ [17], or incorporation of gold nanoparticles [31]. Originally intended to induce an anticoagulative effect, clinically approved oxygenators utilize AH coated HFM, which also turned out to enable effective EC adhesion and formation of a viable, confluent, and functional monolayer under static culture conditions [19]. However, conceivably due to insufficient AH coating, EC detachment was noted to some degree under dynamic flow conditions [20]. Without the opportunity to exactly reproduce the proprietary and confidential AH coating technique, we sought to establish an alternative HFM coating using FN. Being a constituent of the native vascular basement membrane [32,33], FN is a physiologic adhesion molecule for ECs via integrin binding to the RGD sequence [34,35]. Thereby focal adhesion complexes are formed, which activate intracellular signaling cascades to regulate cell spreading, proliferation and cell survival [36,37]. Based on these properties, FN has generally been used to enable sufficient EC adhesion to various hydrophobic materials [22,38,39]. Along with the establishment of our FN coating on the 3D HFM, some significant advantages over the AH coating could be verified. Especially with respect to our coating protocol, homogenous FN coating could be achieved by simply submerging the HFM in FN solution, compared to AH coating, which was described to include multiple, complex dip coating and chemical cross-linking steps [18]. Moreover, unlike the proprietary AH coating, our facile FN coating protocol can be further enhanced and optimized, e.g., by changing FN concentration, or incubation time. Additionally, for future long-term BHL application, FN coating could be complemented by specific EC-homing factors, to attract EC progenitor cells from the blood stream [40] in order to support the regenerative capacity of the monolayer.

Concerning the endothelialization, comparable results under static culture conditions could be shown for monolayers on both coatings. FN coated HFM successfully promoted the formation of a viable EC monolayer, with the same quality and cell density as on AH coated HFM [19,20]. By visualizing the cell-cell junctional protein VE-cadherin, monolayer integrity could be proven, inter alia, as a crucial feature to effectively mask the foreign, pro-thrombogenic HFM surfaces from the circulating blood. Furthermore, in either case, monolayers retained the non-inflammatory and non-thrombogenic state, as no significant differences in the expression levels of the activation-related genes ICAM1, VCAM1, E-selectin and thrombomodulin could be found. Moreover, monolayers on both coatings were also able to reduce the number of adherent platelets to a comparable degree, thereby improving the hemocompatibility of the HFM. However, when deliberately challenged with TNFα ECs on both coatings kept their physiologic responsiveness, which could be detected by the respective gene expression level changes. Interestingly, another advantage of the ECs on the FN coated HFM indicated, that E-selectin upregulation was significantly less prominent compared to ECs on AH HFM, while also the regulation of other analyzed activation-relevant genes seemed less pronounced. Accordingly, a functional adhesion assay revealed a higher number of adhered leukocytes to the TNFα stimulated monolayer established on AH HFM, indicating that ECs on the FN surface may have a higher tolerance towards TNFα treatment. This feature will be beneficial in the clinical BHL setting, where the non-activated phenotype of the EC monolayer needs to be maintained to ensure the hemocompatible surface. However, these results need to be verified in future in vivo studies applying long-term exposure of the endothelialized HFM to whole blood under various dynamic conditions. Another advantage of the FN coating was the coating uniformity. While we detected a homogenous signal for FN all over the HFM surface, AH coating presented several inhomogeneous and non-covered areas. Although it was possible to establish a confluent monolayer on AH coated HFM under static conditions, we hypothesized that endothelial adhesion on the non-coated hydrophobic areas was not strong enough and, besides other impacts mentioned subsequently, entailed the observed cell detachment under flow conditions.

In order to exert flow conditions, we established a BioStat mock circulation, which was designed to simulate BHL relevant, clinical settings [28], imitating various pO_2_ and pCO_2_ blood levels as seen in patients suffering from different lung diseases, or rather different flow dynamics during disease dependent BHL application, e.g., veno-venous or rather veno-arterial [41] cannulation, providing the important pre-translational test platform for BHL assessment under these disease-like conditions. However, the set-up used in this study ensured constant arterial blood parameters under the applied flow conditions, to exclude any detrimental effects on the EC monolayer behavior, e.g., glycocalyx degradation, as seen for example in ischemia and reperfusion injury [42] or rather ROS (reactive oxygen species) production, which occurs during hyperoxemia and adversely triggers pro-inflammatory cell responses or even induces apoptosis [43].

In this study, following flow exposure, ECs were still detectable on both coated HFM, but in relation to the counted cell number before flow application, the higher number of viable ECs remained on the FN coated HFM. Thereby, the level of observed cell detachment from AH coated HFM under flow conditions was at a similar magnitude as detected in earlier studies [19,20]. This increased cell retention was in conjunction with the improved uniformity of the FN coating, homogenously covering the whole HFM surface. Interestingly, immunofluorescence detection for the de novo synthesized collagen type-IV matrix reflected the coating uniformity, as this basement membrane-like matrix appeared evenly dispersed on the FN HFM, in comparison to the patchy and more fibrous matrix structures found on AH HFM, thereby potentially presenting a more physiological niche resulting in improved EC adhesion. However, expression analysis of the steadily expressed COL4A1, a major constituent of collagen-type IV, did not indicate regulation of the collagen type-IV synthesis induced by the surface coating, suggesting that the observed ultrastructural differences were caused by other activated mechanisms, downstream the FN induced signaling.

Besides the homogenous FN deposition, this improved cell adhesion was anticipated with the FN coating, as it is not only a physiologic cell adhesion factor, but also a driver of physiologic cell behavior. One of these positive effects, which was exclusively detected on FN coatings under flow conditions, was the significant upregulation of SDC2, a transmembrane proteoglycan, which contributes to the glycocalyx production [44]. This up to several µm thick extracellular matrix on the blood facing side of ECs [45] is normally not expressed under static conditions [46,47]. Conversely, the glycocalyx has also crucial mechano-sensing functions to mediate shear stress induced signaling cascades into the cells, resulting in cytoskeletal remodeling, control stiffness and height of the ECs [48]. Most importantly, shear stress is also a crucial signal for the upregulation of the anti-thrombogenic surface protein thrombomodulin [49], which also was significantly upregulated in the flow exposed cells of this study. A pivotal transcription factor needed for these shear stress activated cell responses is KLF2 [50], which also was found to be significantly upregulated in this study under flow conditions, however, on both coated HFM. Moreover, such an impregnated endothelial monolayer would also provide an additional barrier function to blood plasma, enabling the use of alternative microporous HFM, which are frequently afflicted with plasma leakage [51]. Thus, the assembly of this endothelial glycocalyx before the exposure of the EC monolayer to blood flow is of importance for BHL application and will be evaluated in future flow adaption experiments, inter alia by our exclusively established cryo-sectioning technique for 3D HFM evaluation. Therefore, this flow adaption protocol will include a basement membrane and glycocalyx-production phase, applying laminar flow with an optimal shear stress, as both factors are described to positively influence matrix assembly [52]. To this end our mock circulation setup can be easily adjusted for the exposure with laminar flow.

Finally, the last positive FN associated effect for both static and flow conditions could be identified by the lower gene expression level of tissue factor in ECs seeded on FN coated HFM. This again underlined the hypothesis that this physiologic endothelial integrin binding to FN mediated an intracellular signaling cascade, causing an alleviated EC monolayer response.

Summarizing, FN coating of the HFM revealed several superior effects regarding the sufficient HFM endothelialization. Most notably, the homogenous FN deposition successfully promoted a viable, confluent, and interconnected EC monolayer, with an improved resistance towards flow conditions, in comparison to the inhomogeneous AH coating. As a component of the native vascular basement membrane, FN induced a more physiologic response of the ECs to external signals, as detected, inter alia, by a significant upregulation of the glycocalyx producing gene SDC2 and the homogenous deposition of the basement membrane collagen-type IV. Additionally, the de novo synthesis of the extracellular matrix will on the one hand secure endothelial adhesion to the artificial HFM, on the other hand reduce cellular resistance to the blood flow, which both support endothelial long-term resistance towards flow application. Both together will provide a further barrier, preventing plasma leakage of the HFM. Moreover, gene expression analysis and functional leukocyte adhesion assay, indicated an increased hemocompatibility of the EC monolayer on FN coated HFM. Based on these results and on the fact that our facile protocol enables homogenous FN coating, this technique will be transferred for endothelialization of additional blood-contacting surfaces, such as housing, cannulae and tubing, which will be geometrically modified from ECMO components to enable the implantation of the BHL, best fitted for the individual requirements of patients regarding the respective anatomic situation and the level of needed lung support. Thus, these FN associated features represent crucial requirements to get one step closer to the implantable BHL as alternative to LTx, which needs to be tested in pre-translational animal models and clinical trials.

## Supplementary Materials

The following supporting information can be downloaded at: https://www.mdpi.com/article/10.3390/membranes12010035/s1, Figure S1: SEM Images for the evaluation of the activation state of the HFM endothelium, Figure S2: SEM images for the evaluation of thrombocyte adhesion affinity to endothelialized HFM, Figure S3: Schematic view of the experimental setup for the application of flow conditions, Table S1: Primer pairs used for the expression analysis of activation-and extracellular matrix-associated genes.

## Abbreviations

AH	albumin/heparin;
BHL	biohybrid lung
DMSO	dimethyl sulfoxide
EC	endothelial cell
ECMO	extracorporeal membrane oxygenation
EGM-2	endothelial growth medium-2
ELD	endstage lung disease;
FN	fibronectin;
HFM	hollow fiber membranes;
LTx	lung transplantation
NC	negative control
PC	positive control
PDMS	polydimethylsiloxane
PMP	polymethylpentene
SBB	Sudan black B
SEM	scanning electron microscopy
TCP	tissue culture plastic
TNFα	tumor necrosis factor alpha
