# Supplementary material for: Towards Biohybrid Lung Development—Fibronectin-Coating Bestows Hemocompatibility of Gas Exchange Hollow Fiber Membranes by Improving Flow-Resistant Endothelialization
Source: Membranes (Basel). 2021 Dec 27;12(1):35. doi: 10.3390/membranes12010035 (PMC8779364; doi:10.3390/membranes12010035)
Supplement: Supplementary file 1 [file membranes-12-00035-s001.zip › membranes-1522431-supplementary.pdf]

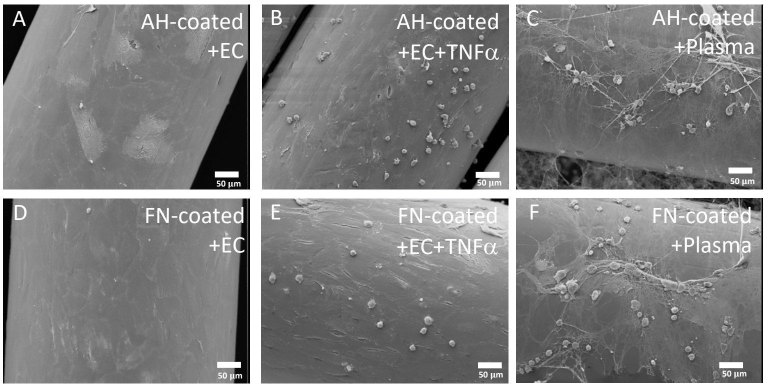

Figure S1: SEM images (A-E) for the evaluation of the activation status of the HFM endothelium. Endothelial monolayers established on the surface of AH (A, B)- and FN (D, E)- coated HFM were either cultivated under standard conditions (A, D) or deliberately challenged with TNFαfor 6 h (B, E) before HL-60 leukocytes were added. While few to none HL-60 cells adhered to the endothelial monolayer under standard conditions (A, D), numerous HL-60 leukocytes adhered to the TNFα activated monolayer (B, E). Blood plasma-immersed, AH-coated HFM (C) and FN-coated HFM (F) served as positive control and resulted in adhesion of significant numbers of HL-60. Scale : 50 µm.

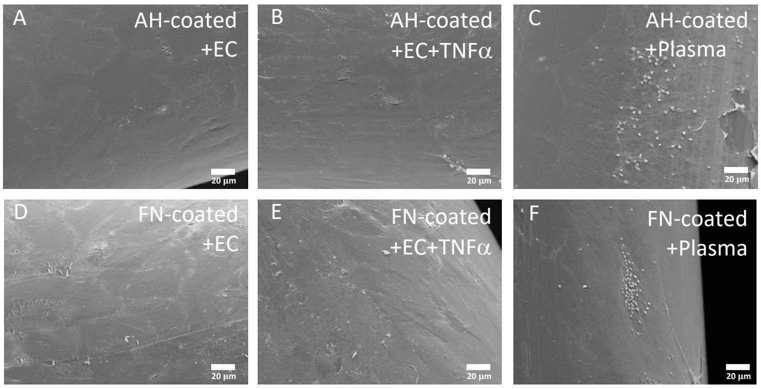

Figure S2: SEM images for the evaluation of thrombocyte adhesion affinity to endothelialized HFM.

Endothelialized AH- (A, B) and FN- (D, E) coated HFM were incubated with thrombocytes to assess the thrombogenicity of the substrate. AH-coated (C) and FN-coated (F), but non-seeded blood plasma immersed HFM served as a positive control. Few to none adhered thrombocytes were found on endothelialized HFMs cultivated under standard conditions (A, D). The stimulation of the EC monolayer established on both AH- (B) and FN- (E) coated HFM with TNF◎ for 6 h did not result in significant higher numbers of adhered platelets. A high number of adhered thrombocytes was found on the positive controls (C, F). Scale 20 µm

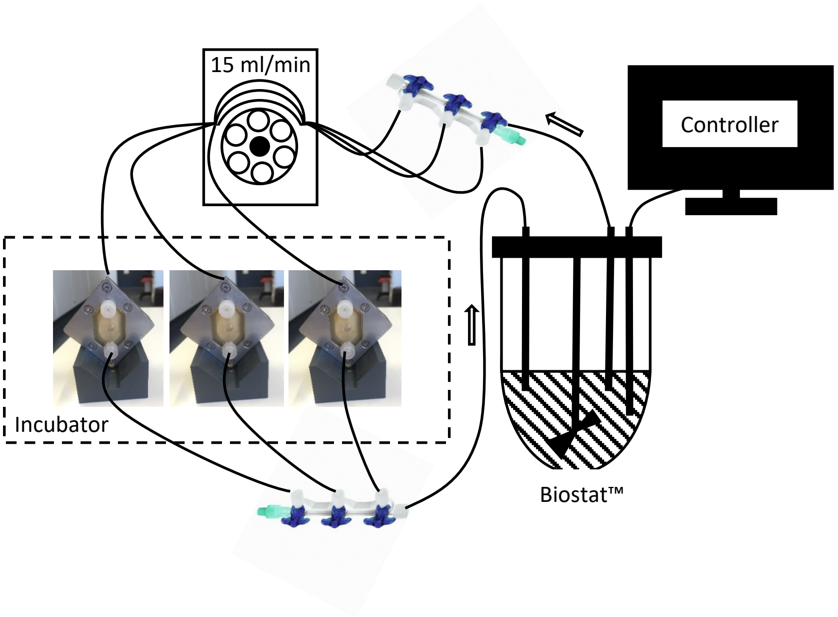

Figure S3: Schematic view of the experimental setup for the application of flow conditions. Medium was warmed to 37°C and adjusted to pre-set gas saturation levels in the stirred tank of the BioStat (Sartorius, Melsungen) system. Medium was recirculated through the tubing and the custom made HFM flow chamber using a roller pump (Ismatek). Three-way manifolds allowed the run of three technical replicates in parallel.

Table S1: Primer pairs used for the expression analysis of activation- and extracellular matrix-associated genes.

Gene	Primer pair sequences (5'-3')	Amplicon length [bp]
ß Actin	ATT GCC GAC AGG ATG CAG AA	176
	GGG CCG GAC TCG TCA TAC TC	
ICAM-1	CTA CCT CTG TCG GGC CAG GA	132
	AGG CCT GCA GTG CCC ATT AT	
Tissue Factor	CCC GAA CAG TTA ACC GGA AGA	191
	GGA GTT CTC CTT CCA GCT CTG C	
Thrombomodulin	GCC CAT GGG AGC TGG TTA GA	190
	GGC CTG ACT TGG CCT GCT AC	
VCAM-1	GGC GCC TAT ACC ATC CGA AA	156
	GAG CAC GAG AAG CTC AGG AGA A	
E-Selectin	ATC CAG CCA ATG GGT TCG TG	114
	GAA GGC TCT GGG CTC CCA TT	
KLF2	CGC ACA CAG GTG AGA AGC CC	196
	GGG CGT CCC GGC TAC ATG T	
Collagen alpha-1 (IV) chain	ACC AGG ACC CAG AGG CAA AC	178
	TCC CAA AGG TCC TGT GCC TA	
Syndecan-2	TCG GCG GAG TCG AGA GCA GAG	127
	CTC CCG AGC CAG ACG CAG AA	
